# Cross-Sector Collaboration to Improve Access to Community Services for People Living With Diabetes: Contributions From Actor-Network Theory

**DOI:** 10.1177/11786329231222408

**Published:** 2024-01-27

**Authors:** Géraldine Layani, Alexandre Tremblay, Marie-Thérèse Lussier, Isabelle Godbout, Hélène Bihan, Claire Gosselin, Mégane Pierre, Aude Motulsky, Isabelle Brault, Isabel Rodrigues, Janusz Kaczorowski, Marie-Claude Vanier, Sopie Marielle Yapi

**Affiliations:** 1 Department of Family Medicine and Emergency Medicine, Faculty of Medicine, Université de Montreal, Montreal, QC, Canada; 2 Research Centre of the University of Montreal, Montréal, QC, Canada; 3 Pôle 1, Centre de recherche des pratiques cliniques et organisationnelles du Centre intégré de santé et de services sociaux de Laval, Laval, QC, Canada; 4 National School of Public Administration, Montréal, QC, Canada; 5 Avicenne hospital, Bobigny, France; 6 Health Education and Practices Laboratory, Université Paris 13, Paris, France; 7 Faculty of Nursing, University of Montreal, Montreal, QC, Canada; 8 Faculty of Pharmacy, Université de Montréal, Montreal, QC, Canada; 9 Department of Management, School of Public Health, Université de Montréal, Montreal, QC, Canada

**Keywords:** Intersectoral collaboration, partnership, care pathway, chronic disease, diabetes

## Abstract

Diabetes is a global public health issue. The Public Health Agency of Canada published a Diabetes Framework 2022 which recommends collaborative work across sectors to mitigate the impact of diabetes on health and quality of life. Since 2020, the INMED-COMMUNITY pathway has been implemented in Laval, Québec developing collaboration between healthcare and community sectors through a participatory action research approach. The aim of this article is to gain a better understanding of the INMED-COMMUNITY pathway implementation process, based on the mobilization of network actor theory. Qualitative analysis of semi-structured interviews conducted from January to March 2023 with 12 participants from 3 different sectors (community, health system, research), were carried out using actor-network theory. The results explored the conditions for effective intersectoral collaboration in a participatory action research approach to implement the INMED-COMMUNITY pathway. These were: (1) contextualization of the project, (2) a consultation approach involving various stakeholders, (3) creation of new partnerships, (4) presence of a project coordinator, and (5) mobilization of stakeholders around a common definition of diabetes. Mediation supported by a project coordinator contributed to the implementation of an intersectoral collaborative health intervention, largely due to early identification of controversies.

## Introduction

The World Health Organization (WHO) recognizes diabetes as a major public health issue.^1^ Diabetes is a widespread chronic disease in Canada which is primarily managed in primary care and integrated into chronic disease management.^2-6^ Over 3 million Canadians, or 8.9% of the adult population, have been diagnosed with diabetes; this is expected to increase by 12% by 2031.^7^ This increase is due to an aging population and an increase in co-morbidity, leading to a significant rise in healthcare expenditure.^8,9^

Living with chronic disease requires flexible integrated care involving various and well-coordinated care and services, from prevention to end-of-life care.^10-12^ An intersectoral collaborative approach is fundamental to meeting ongoing needs and achieving this integrated care.^13^ According to the Public Health Agency of Canada’s 2022-27 Framework, the management and control of diabetes in Canada requires intersectoral collaboration with stakeholders from public, private, and non-governmental sectors to ensure an inclusive and comprehensive perspective on the social determinants of health, as well as a person-centered approach.^9^ Studies on intersectoral actions for people living with diabetes have pointed out how the implementation process can be effective, but few have looked into the impact of intersectoral collaboration on people health.^14,15^ Social support systems that focus on the complex empowerment processes of people living with chronic diseases can also reduce social inequalities and inequities and impact of diabetes on health.^9^

Intersectoral collaboration is a well-established strategy for mitigating complex societal problems and addressing systemic changes.^16,17^ Intersectoral collaboration can create interconnectivity between different systems (health, socioeconomic, and environmental), to promote comprehensive interventions that address both individual needs and population health.^18,19^ Different intersectoral initiatives addressing the determinants of health can link public policy to better population outcomes.^20^ The present study is based on the WHO definition of intersectoral collaboration in health stated in Nuthbeam and Muscat^21^: “intersectoral collaboration in health constitutes all actions undertaken by different sectors of society to achieve health outcomes in a more effective, efficient or sustainable manner than if one sector worked alone”.

### Actor-network theory (ANT)

This study is based on the theoretical framework of actor-network theory (ANT). This theory was developed by sociologists Madelaine Akrich, Michel Callon, and Bruno Latour and recently adapted by Bilodeau and Potvin^22-27^ to empirically describe and analyze networked action in public health interventions.^28^ This framework is adequate in an organizational context where multiple stakeholders from different sectors and social groups are brought together, form alliances and develop ways to act in a coordinated manner.^29^ ANT conceptualizes networked action as a process involving human and non-human actors (eg, specialized knowledge, resources, technologies) linked by a problematic situation.^25^ Consequently, ANT is used to better understand how sociotechnical networks produce change,^30^ through creation, reconfiguration, and extension of the network in which new solutions emerge in response to a shared problematization.^26^ Callon and Latour refer to this process as “translation.”^22,25^ According to Akrich et al and Bilodeau et al, “translation” is a process made up of 5 overlapping phases: contextualization, problematization, interessement, enrollment, and mobilization.^23,26^ Contextualization enables the effects of social innovations to be measured by the actors and their actions understood in context.^31,32^ Problematization leads actors to develop a shared vision, rallying them around a common interest. Interessement refers to actions to encourage new stakeholders to join a project, adhere to common goals, and participate in its realization. Enrollment refers to the negotiation and acceptance of new roles and mobilization refers to the effective realization of connections between actors who can act as spokespeople for the network and generate action.

### INMED-COMMUNITY pathway

The INterprofessional Management and Education in Diabetes care (INMED) pathway^33^ is an participatory action research (PAR)^34,35^ that emerged from the recommendations of Diabetes Canada^36^ and the principles of the Revised Chronic Care Model^37^ addressing people living with diabetes in Laval, Quebec. For its part, the INMED-COMMUNITY pathway focuses on development of intersectoral collaboration between the healthcare and community sectors to improve accessibility of community services for people living with diabetes.

The INMED-COMMUNITY pathway developed intersectoral links within a network of actors and complex actions. The 5 main aspects of ANT (contextualization, problematization, interessement, enrollment, and mobilization) were used to analyze the intersectoral collaboration of the INMED-COMMUNITY pathway: (1) restructuring (contextualization), (2) consultation approach (problematization), (3) creation of new partnerships (enrollment), (4) project coordination and joint definition of diabetes (interessement), and (5) alignment of actors, resources, and network support (mobilization). This analysis is based on the modeling of ANT and intersectoral collaboration by Bilodeau et al.^26^

The aim of this article is to gain a better understanding of the INMED-COMMUNITY pathway implementation process, based on the mobilization of network-actor theory.

## Method

### Study estimate

The study is based on an analysis of the development of intersectoral collaboration between health and community sectors of people living with diabetes as part of intervention implementing the INMED-COMMUNITY pathway in Laval, Quebec. The team has been involved in a PAR^32^ to facilitate the implementation of the INMED-COMMUNITY pathway in two university family medicine groups (U-FMGs) in the Laval region (Quebec) since April 2022. PAR is particularly appropriate for our research, as it relies on the involvement of health organization and community stakeholders at all stages of the research^38^ and encourages action through the creation of spaces where stakeholders can identify needs and propose solutions.^39,40^ Actors involved in the INMED-COMMUNITY pathway are from health, community, and research sectors (Table 1). Since April 2022, stakeholders have been invited to participate in the process of implementing the INMED-COMMUNITY pathway through formal and informal weekly meetings led by the research team, and through committees whose frequency varied according to the project objectives to be achieved (monthly to quarterly).

**Table 1.** Characteristics of professionals involved.

Sectors	Specialties	Primary care sector
Health care system	Primary care	FMG
		CISSS
		N/A
	Specialized care	N/A
Community	Public health	N/A
	N/A	N/A
Research	N/A	N/A
Participants	Categories	Sectors
Actor 1	Quality improvement officer (professional)	Healthcare system
Actor 2	Patient-partner	Community
Actor 3	Primary care manager	Healthcare system
Actor 4	Primary care manager	Healthcare system
Actor 5	Primary care manager	Health care system
Actor 6	Executive director of a community organization	Community
Actor 7	Specialized care nurse	Healthcare system
Actor 8	Primary care nurse	Healthcare system
Actor 9	Research project coordinator	Research
Actor 10	Research project coordinator	Research
Actor 11	Primary care manager	Healthcare system
Actor 12	Community organizer	Community

### Recruiting participants

Qualitative exploratory research was conducted between January and March 2023 through semi-structured interviews with 12 participants from 3 different sectors (health, community, research) recruited using a purposive sampling process (Table 1).^41-43^ The criterion used for recruitment was to have participated in the implementation of the INMED-COMMUNITY pathway. This approach recorded the perspectives of different actors involved in the PAR intervention in the INMED-COMMUNITY pathway. Recruitment was done to represent all stakeholders that took part in the INMED-COMMUNITY pathway. Overall, 3 participants were from the community sector, including a patient-partner, a director of a local community organization and a community organizer. Two participants served as project coordinators working with the research team. Seven participants represented different stakeholders from the health sectors: 2 nurses, 4 health administrators and one quality improvement agent.

### Study process (action/reflection cycles)

As described by Cargo and Mercer,^44^ the PAR approach was based on 3-phase cycle systems: (1) analysis of the situation and the change to be achieved by the actors (research), (2) joint analysis of results and feedback, and (3) reflection on the results of data collection, planning and implementation of the change to be achieved (action). These phases were repeated over several cycles for each action that engaged stakeholders in the implementation of the INMED-COMMUNITY pathway.

The actions of the INMED-COMMUNITY pathway were determined in collaboration with all stakeholders and approved by the multisectoral representatives through an iterative process that followed 6 steps: (1) in June 2022, a brainstorming workshop was organized with all stakeholders so they could get to know each other, initiate collaborative links and clarify their service offers in order to become more familiar with the service offers available in the health and community sectors, (2) in July 2022, a “resource directory” was suggested by one of the patient partners, as a useful and relevant tool for monitoring people with diabetes. This suggestion was discussed and appreciated by the other stakeholders at a project meeting, who collectively adopted the idea and set up a “resource directory committee” to ensure co-creation of the “resource directory” and integration of all participants’ ideas, (3) in October 2022, a “self-care support guide” for people living with diabetes was discussed and adopted by all participants of the “resource directory committee” to better support people living with diabetes and self-managing the disease, (4) in August 2022, participants discussed the importance of creating a form to refer patients from the clinical sector to the community sector. This idea underwent several adjustments and was finally validated and adopted in September 2022 by all the participants, (5) in November 2022, an expanded working committee was created to reflect on the organizational anchoring of this project and the terms of commitment of all stakeholders in the sustainability and 6) in February 2023, a project for an intersectoral table committee emerged following a consultation of all stakeholders to contribute to the sustainability of the INMED-COMMUNITY pathway in the organization. These cycles enabled the gradual implementation of the INMED-COMMUNAUTE pathway, which is still ongoing.

### Data collection strategy

A qualitative interview guide (Appendix 1) was developed based on actor-network theory.^26^ Interviews lasting approximately 45 to 60 minutes, were conducted by 3 members of the research team via an online video conferencing platform, and then were transcribed. The research team members conducing the interview were distinct from the research coordinators interviewed for the study. Field notes were also taken at various meetings and project activities. The principal investigator (GL) kept a logbook to document her understanding of the phenomenon under study. The elements collected provided multiple data sources that were triangulated to reach data saturation.^45^ Data saturation was obtained throughout the analysis as no new code emerged.^46^

### Analysis

A deductive and inductive qualitative thematic analysis^47^ in double coding was carried out using NVivo software^48^ and informed by the ANT framework.^25-27^ Five interviews were coded by 2 members of the research team, to create an initial code tree. A team meeting enabled the code tree to be adjusted based on the 5 main aspects of the ANT framework. The analysis of all interviews was then done by one of the team members (AT) using the agreed codebook.^49^ Inductive coding continued after the initial codebook had been developed, and new codes emerged. Preliminary results from the data analysis were discussed in a team meeting to validate any adjustment needed. Data from the interviews were triangulated with field notes taken during meetings and committees with stakeholders and the logbook notes of the lead researcher (GL).

## Ethical process and consent to participate

The organization’s ethics committee has given a favorable opinion on the original study (2020-602).

## Results

Participant characteristics are presented in Table 1. Three participants were from the community (a patient, an executive director of a community organization and a community organizer), 4 were health system managers (primary care) and 4 were primary and specialized care professionals. Analysis of the qualitative results explored the conditions for achieving intersectoral collaboration in an actor-network theoretical approach for people living with diabetes by mobilizing the 5 main aspects of ANT.^22-27^ In addition, the implementation of the INMED-COMMUNITY pathway through PAR identified several elements that facilitate collaboration between people from different sectors (community and healthcare). In particular, the meetings held between all the participants from different sectors enabled them to listen to each other and adjust throughout the course of the research. Although people from the community were in minority, the stakeholders ensured that they were listened to and well represented during the meetings. These were grouped according to the 5 components of ANT: (1) restructuring (contextualization), (2) consultation approach (problematization), (3) creation of new partnerships (enrollment), (4) project coordination and common definition of diabetes (interessement), and (5) alignment of actors, resources, and network support (mobilization).

### Contextualization

The results of the interviews demonstrate how participatory action research was conducive to the restructuring of each system involved. First, as one of the primary care managers mentioned, COVID-19 fostered teamwork and enabled the stakeholders to “work together on a common project” (Table 2, extract 2). Another primary care manager also mentioned that policies had been favorable to the development of the INMED-COMMUNITY pathway, given that this project “fits in” with current ministerial priorities (Table 2, extract 3). Finally, the community organizer mentioned the implementation of another trajectory project, which in their view facilitated the stakeholders’ adherence to the INMED-COMMUNITY pathway (Table 2, extract 5). On the other hand, the “siloed” organization of policies was perceived as a barrier to restructuring according to a specialized care nurse (Table 2, extracts 6 and 9). Moreover, according to a primary care professional, reduced funding for community organizations led them to reconsider their activities (Table 2, extracts 7 and 8). These results are corroborated by the field notes, which emphasize the collaboration between managers from different departments and sectors working together to overcome the difficulties of the COVID-19 context.

**Table 2.** Contextualization.

Themes	Subtopics	Verbatim
An environment conducive to restructuration	Covid-19	[. . .] ***the advantage of the pandemic is that it brought us 4 more positions***, *we didn’t have much development, it takes a long time to develop community organizer positions, but the pandemic helped us develop 4 positions.* (Extract 1_Actor 12) *Yes, because* ***before the pandemic, we were much more separated***, [. . .] *to say* ***that we were working together on a clinical scope*** *or on a clinical subject together, our teams together*, ***it was our first time there with the diabetes research project***, *so it set up a very nice alliance.* (Extract 2_Actor 5)
	Policy	*It’s a big difference. In the current context*, ***I think it fits in so well with the Ministry’s vision of the projects to be set up***, *us starting over, then starting again, but knowing that everything still needs to be done, and then we’re also waiting for the Ministry’s recommendations, in terms of what it wants, where it wants each of the regions to head for a population-based service offer, because right now our population-based offer is virtually non-existent in (city name).* (Extract 3_Actor 3)
	Organizational	*Because* ***I experience it in other types of things that I work on where we really work inter-directionally***, *and there’s no problem there, because it’s established, it’s known, each of the actors from each place is there, we meet, and that makes it works. It’s just a matter of putting it there, but I think that*, ***you know, chronic illness should be a priority, the first line***. (Extract 4_Actor 4) *Well, it’s true that, as with family caregivers, there are often more referrals*, ***with the Alzheimer’s Society, there’s a trajectory that’s already defined***, *that was developed in (city name), but they’re in the process of deploying throughout Quebec, an Alzheimer’s trajectory, a bit like here with diabetes, the person who is diagnosed with Alzheimer’s is automatically referred to the Alzheimer’s Society in the territory.* ***This is already done in (city name), and it’s established, but it’s managed by SAPA*** [. . .]. (Extract 5_Actor 12)
Obstacles to restructuration	Policy	[. . .] *but at the Ministry, it’s someone who’s more of a hospital type, not primary care, so* ***all these people at the top need to talk to each other, because that’s what we were saying, it’s something that’s not usual, from what I’ve understood, at the Ministry, they say we shouldn’t work in silos, but they themselves are quite siloed*** *[RR], so they need to look in their own backyard too.* (Extract 6_Actor 7)
	Economic	*I spoke to the Ministry on Tuesday and said, “You say you have $200,000 for all general social services, mental health, that’s because I need 3 people just for us, with my social workers and the U. I don’t even have anyone on my team to do that. I don’t have anyone on my team to do that*, ***my clinical coordination isn’t even funded, I fund it out of my absences, so how do you expect me to. . . you know? It’s a provincial issue.*** (Extract 7_Actor 11) [. . .] *then we’re sensitive to that too, we’re more and more on the lookout for* ***what the community is doing, and you know, they have funding issues, and it’s not easy running a community organization***, *so the more references they have, the more we know them, but it seems like they don’t dare come knocking on our door.* (Extract 8_Actor 1)
	Organizational	*Well, that’s it, it becomes* ***a lot of operating methods which are different for many reasons, and that have been developed in silos***. *But that’s precisely the same essence of saying that professionals are more involved in case management, and that the doctor becomes the second level of intervention, when there’s a particularity, a medical adjustment* [. . .] ***and it’s a constant collaborative effort between all these people.*** (Extract 9_Actor 1)

### The consultation approach

Results of the interviews show that consulting stakeholders through formal and informal meetings and committees helped create new links in the INMED-COMMUNITY pathway. The interviews and field notes highlight that the consultation approach facilitated: (1) meeting of new stakeholders, (2) presentation of different service offerings, (3) creation of links between different sectors that did not collaborate together before the implementation of the INMED-COMMUNITY pathway, (4) perception of the complementary roles of all stakeholders involved in the project, (5) anticipation of new needs to implement the INMED-COMMUNITY pathway, (6) development of new ideas and associated know-how to implement it, and (7) positive spin-offs for patients.

However, this consultation-based approach required favorable conditions to be operational and engage all stakeholders toward a common goal, as mentioned by several different types of participants (Table 3). One of the primary care managers mentioned the importance of “trust” and the adoption of a “common language” to be able to work together (Table 3, extract 1). One of the project coordinators also mentioned the importance of sharing common “values” and “changing the beliefs” of those involved to be able to mobilize them during consultations (Table 3, extract 5). These observations were supported by another manager and a primary care professional, who added that sharing values had required a certain “humility” on the part of the participants (Table 3, extract 9) and required questioning “relevance for patients,” to be able to adopt a common vision of the INMED-COMMUNITY pathway (Table 3, extract 13).

**Table 3.** Problematization.

Themes	Subtopics	Verbatim
The consultation approach facilitates links between new actors	Meet new actors	[. . .] *when we talk about interdisciplinarity at the clinical level, or about interdisciplinary collaboration or trajectories with other sectors, the* ***most difficult thing is to establish trust between each of the members***, *trust that the work will be well done, trust that we know each other’s service offer well, trust* ***that we have a common language to get there***, *trust that together we’ll have an impact on the customer*, ***and trust that the customer will be part of the solution***. *For me*, ***it’s the link***, *then it’s* ***the dynamic between the actors***, *apart from knowing the actors, because no matter how well I know the actors*, ***if together we don’t see ourselves as part of an even bigger whole, that’s where we’re missing the boat***. (Extract 1_Actor 3) *I think this was one of the great strengths of the research project, not in the past tense, but in* ***the*** *present tense, because* ***to have been able to go and find all these actors***, *to* ***get them around a table*** *and* ***discuss things together***, *then to see if the different ways of seeing, the different ways of doing things from one and the other, I think that yes, quite rightly, people were called upon and were called upon constructively.* (Extract 2_Actor 5)
	Presentation of the different service offers	[. . .] *there’s* ***knowing the other’s service***, *knowing what they offer, what our place is*, [. . .] ***so we channel our energy into something else***, *then* ***we refer to our partner, which is Association (name of association) for its mission, for which it is subsidized***. (Extract 3_Actor 1)
	The creation of links between different sectors that did not work together before the implementation of the INMED-COMMUNITY pathway.	[. . .] ***It’s not natural for healthcare to work with the community*** *[R], social services do work with the community, but healthcare doesn’t necessarily, it’s not necessarily natural. That’s a* ***good thing about this project***, *because it’s not just a top-down approach, and it’s not just cheap dispatching.* (Extract 4_Actor 12) [. . .] *we confronted our partners and made them realize that, in fact*, ***collaborative work is possible***, *we have things to bring to each other, so the main spin-off, for me, is* ***the mobilization of people’s beliefs***, ***the change in beliefs that*** *will certainly occur, in my opinion. They’re going to start working together, they’re already starting to work together through the referral form, but that’s going* ***to lead to changes in their values for greater openness, then greater collaboration***. (Extract 5_Actor 9) [. . .] *I thought it was really great and innovative, because it* ***’s very rare that we have community organizers, representatives of associations***, ***second line***, ***first line***, *then it continued to evolve* [. . .] *then* ***that’s a big strength that I would keep***, *because it* ***helped us to have the mobilization*** *and the possible with* ***community partners***, *then it’s by working on projects like that that we* ***develop the personal link too***. (Extract 6_Actor 1)
	Perception of complementary roles of all actors involved in the project	*I could certainly see that a* ***huge number of people had been consulted and involved***, *whether in the meetings we’ve had more recently or even in the presentations we’ve had, we could see that* ***all the pieces had been attached with the relevant people.*** [. . .] *there had been* ***an attachment*** *with lots and lots and lots and lots* ***of different perspectives***, ***different resources***, *people from academia, people from practice, people from the community, we saw all that represented, really* ***a very interesting panoply***, ***links with environments in other regions***. *So I think that was. . . we* ***really felt that it was important to have the perspective of everyone affected by this***. (Excerpt 7_Actor 4) *With the progress that everyone has made in the last year, at the level of the participants, we’ve perhaps realized that yes, there are* ***things that fit together***, *there are things that have grown in scope, there are files that are a little more relevant, there are others that have been a little put aside or that have been modified to bring them. . .* (Extract 8_Actor 6)
	Anticipating new needs to set up the INMED-COMMUNITY pathway	[. . .] ***When I said it couldn’t be done, well, we abandoned that idea, and I thought that was really nice, because it’s the kind of clinical humility you have to have***, *and then say “OK, we thought it up like that, but I don’t think it will work for a lot of factors in the field, that* ***we readjust right away instead of persevering with an idea that won’t work in the long term***, *or that may not give us the results we’re really hoping for in terms of sustainability,” I thought that was really good*. (Extract 9_Actor 1)
	The development of new ideas and associated know-how to put the trajectory in place	*Yes, the good you do to the people who participate in your project*. ***The people we’ve dealt with*** *have always been polite, and no one has ever said a bad thing to each other while we’ve been there. It’s like you’ve targeted a problem, and then made sure we could find solutions to that problem* [. . .]. (Extract 10_Actor 2) *When we started meeting with people from CLSCs and FMG, I began to see* ***that this was pointing to a problem that everyone was aware of, and as time went on***, *taking part in these meetings made me think differently too, I thought, “How can I help them, how can I make my collaboration useful?* (Extract 11_Actor 2) [. . .] *I saw* ***a convergence of objectives that made it possible. . .*** *I saw* ***the possible application in various environments*** [. . .] *throughout the project, I was able to* ***appreciate the advances*** *and then the* ***paradigm shifts***, *the* ***changes in approach***, *we were all on the same level, on the same wavelength, I saw* ***the*** *quiet* ***continuity we wanted to establish*** [. . .]. (Extract 12_Actor 3)
	Benefits for patients	*It’s a fine example of, yes, we’re getting out there, but at the same time it shows exactly how all this partnership, and the links that have been created, lead to. . .* ***then in the end, it’s for the patients that we’re going to have an offer that’s more interesting***, *because we’ve got out there, we’ve seen our thinking differently, bringing in new points, new perspectives that we wouldn’t have achieved if we hadn’t had these exchanges.* (Extract 13_Actor 4) *It’s also good for me, you know. I was able to attend a cooking class, I went to numerous meetings organised by (name of association) association, so I think it gives me additional information. It’s not true that diabetes is tragic, if we are wise and we understand what is happening to us, we are more capable to collaborate on this* [. . .]. ***To know why, you need to have information, you need to have education, you need to have lots of things that help you, and that was not available before.*** *If now when in contact with new diabetic patient* ***we can give them all this information, then I think it will make them better patients.*** *In the end, it will save money, it will save time, and it will improve the well-being of people.* (Extract 14_Actor 2)

### Creating new partnerships

The implementation of the INMED-COMMUNITY pathway has furthered the creation of several partnerships within the health sector (inter-directorate), with the health sector and the community sector (intersectoral), and between the INMED-COMMUNITY project team (research sector) and other external participants (peripheral), as described in the INMED project implementation field notes. Partnership with patient partners was perceived by most actors as central in justifying their commitment to intersectoral action.

#### Interdirectorate partnerships

Analysis of the field notes shows that PAR activities have furthered the creation of new collaborations between the various actors from different departments. The involvement of new actors in the INMED-COMMUNITY pathway enabled the mobilized actors to rethink the organization of primary care for people living with diabetes differently at health sector level, as mentioned by a manager and a primary care professional (Table 4, extract 1). Most managers also mentioned the value of “limiting duplication of services” by pooling professional resources in primary care to organize care and services for people living with diabetes (Table 4, extracts 2 and 3).

**Table 4.** Interessement.

Themes	Subtopics	Verbatim
Create new partnerships	Inter-directorate	***The partnership with the community organizers***, *whom we knew, who were part of the CISSS (region name)* ***but were in a completely different and somewhat unknown position***. [. . .] *What I’d like to see at some point is* ***a community organizer*** [. . .] ***who would be responsible for setting up this mechanism for sustainability***, *and for* ***saying that it’s its job to validate the offer of services in diabetes with the secondary care, the primary care and then the community organizations***, *and that we meet frequently* [. . .]. (Extract 1_Actor 5) [*I think there was a* ***lack of knowledge*** *that led to a* ***lack of coherence in the services***, *then* ***duplication***, *I think that the main knot that was untied was to create that link*, ***then the communication bridge between the two*** *[. . .*]. (Extract 2_Actor 1) *There’s also the whole notion, that we must not forget, of* ***not duplicating*** *services. If XX aims to teach pre-diabetics, that’s not part of the clinical nurse’s service offering, it’s part of the FMG nutrition service offering, so the message is to refer to XX, XX is in our trajectory.* (Extract 3_Actor 6)
	Intersectoral	*Basically, for me*, ***my main partner is XX***, *in the long term, with whom I’ll be keeping in touch, that’s for sure* (Extract 4_Actor 12). *I* ***know now that the Association (name of association) is (name of the executive director of a community organization)***, *and that if it has groups, I’ll be tempted to share them with my colleagues because* ***I know how they work***. *The fact is that this is the kind of business that develops when you’re working on projects, and you don’t have many opportunities to get to know them and see them in person, made* ***this a really big, big strength that I appreciated***. (Extract 5_Actor 1)
	Peripheral	[. . .] *but you also had this role of facilitator, of* ***linking with other regions, sharing good practices, best practices which are in place in other environments***, *which we don’t often have access to because we’re very small sector still in silos. So, we called on the expertise of another sector that had set up a great project, and they were very warm, very generous with their project, with their. . .* ***their intellectual property, they have shared all their documents, they’re going to use clinical performance indicators that they’re ready to share with us*** [. . .]. (Extract 6_Actor 3)
	Patient-partner	*Yes, and there are two ways in which this benefits us: there’s the diabetes aspect, of* ***always having the partner patient’s monitor how she’s living***, *what exactly she’s been through*, ***it’s experiential knowledge which is extremely enriching when we think about how we align our care and then our services*** *[. . .*]. (Extract 7_Actor 5) *In the various consultations, we work with* ***patient-partners***, *sometimes, when it’s managed by program directors, I think that it takes* ***us out of our theory, our practice, out of the matters that’s our business*** [. . .] *for me*, ***they’re the most important***, [. . .] ***they’re the ones who experiences it on a daily basis***, *then* ***who have problems with the health network or with the community network***, *so* ***they’re the ones we have to listen to first***. (Extract 8_Actor 12)

#### Cross-sector partnerships

All stakeholders expressed a willingness to create partnership links between the community and healthcare sectors. The community organizer mentioned the relevance of calling on community participants to respond to person-centered needs and stated “the potential to perpetuate” these links to work together over the longer term (Table 4, extracts 4 and 5).

#### Peripheral partnerships

The field notes described the relevance of the collaboration created by the INMED-COMMUNITY project team and a team of professionals from another region of Quebec, who held tools useful for educating and supporting the self-management of people living with diabetes online. This peripheral collaboration was understood by one of the primary care managers as an opportunity to “share expertise” to “enrich the care offer” available to people living with diabetes (Table 4, extract 6).

#### Patient partnership

All the participants mentioned that the “experiential knowledge” of the patient partners had been indispensable in linking theory and practice, and in reflecting on useful organizational processes for patients; this was present both in interviews and in field notes taken by the research team. One of the managers justified the involvement of patient partners as a contribution to clinical “good practice” and research. Participants were swayed by the importance of “listening to patients first” to enable innovative organizational interventions that can meet their needs, as reported by the community organizer (Table 4, extracts 7 and 8).

### Involving stakeholders through project coordination and a shared definition of diabetes

The analysis shows that the partnership with the research team was established through recognition of the role of the 2 INMED-COMMUNITY project coordinators. Their main contribution revolved around controversies arising at meetings and committees. The principal researcher’s field notes show that links established with participants enabled gradually identification of the project’s controversies, explored how to facilitate the evolution of action, and contributed to the formation of network through the realization of various selected deliverables.

One controversy raised during the INMED-COMMUNITY pathway was linked to the fear of duplication for preventive and educational services between a local community diabetes organization and the health sector. This controversy was addressed during the INMED-COMMUNITY pathway in a working reunion where stakeholders from both sectors could share information about their respective services. Subsequently, the local community diabetes organization was invited to deliver 2 presentations about their services within the participating U-FMGs. This initiative aimed to streamline the process of health providers referring diabetes patients to the local community organization.

Despite controversies identified by the principal researcher, they were not raised by participants in the interviews. In fact, most of them spoke of the coordinators’ “facilitating” roles in creating links and engaging in actions (Table 5, extracts 1 and 2). A primary care manager also mentioned that presence of the research team had facilitated the “transformation of practices” desired by the implementation of the INMED-COMMUNITY pathway, and that it had supported the transformation process (Table 5, extract 3). The “pro-active involvement” of the research team was also highlighted by one of the professionals as a facilitating factor for collaboration and its “sustainability” (Table 5, extracts 3 and 4). One of the patient partners mentioned the importance of managers in “gathering ideas, removing what was not useful and keeping the interesting content of exchanges” to create value (Table 5, extract 5). One of the managers mentioned that this role had the potential to be “transferred” to another team to perpetuate the implementation of the INMED-COMMUNITY pathway (Table 5, extract 8).

**Table 5.** Enrollment.

Themes	Subtopics	Verbatim
Presence of project coordination	Facilitators	*Basically, for me, it was more of a facilitating role* [. . .]. *For me, the word “facilitator” is important, because the fact of having* ***a special time to share and explain*** *with (co-investigator principal) and (research project coordinator) enabled me to* ***fully understand your project, and*** *then to make sure that my team members were involved, that they* ***knew my position so that they could participate in the project, and then see to what extent they could get involved***. (Extract 1_Actor 4) *It’s like what I generally do with research projects*, i.e. *I become a* ***sort of facilitator between the research team and the reality in the field***, *so that I’m not the one coordinating, in charge of procedures and so on* [. . .]. (Extract 2_Actor 1)
	Proactive involvement	[. . .] *And* ***you’ve been very kind, very patient***, *I think, in* ***helping me understand a research project aimed at transforming practices***, *because* ***the word “research,”*** *in my head as a manager*, ***stuck to fundamental research***, *more [R] whereas when we’re talking about transforming practices, that’s something completely different.* ***I’d like to thank you for your patience, because it took me a while to understand*** *that it wasn’t you who was coming into my environment, but rather I who was calling out to you* [. . .]. (Extract 3_Actor 5) [. . .] *I really appreciated that*, ***that we were consulted a lot, I learned from that too, then I found it super interesting as a way of operating*** [. . .] ***you were there in the environment, you came to understand, you came to the meetings often, you met the management committees, you know, it was a proactive involvement*** *[. . .*]. (Extract 4_Actor 1)
	Idea sorting	*It’s very important because they also have different experiences and they brought an interesting vision, and it was fun because I had the impression, in any case, that (co-investigator principal) and (research project coordinator) worked well together. Yes, I think they’re both very important*, ***they were the ones who brought together all the ideas, then removed what wasn’t useful, then kept what was interesting***. (Extract 5_Actor 2)
	Promote project sustainability	*That’s what I’m hoping to create, and we’re also in the process of* ***talking about it with people higher up in management***, *because I think that for all this to last, yes, it needs to last over time, and yes*, ***it needs to continue to have one person who has a greater oversight of what we’re all doing together, to avoid duplication of services***. *Or at some point, there’s also a risk that we’ll think that the neighbor or community organization is doing it, and then we’ll stop providing it, or vice versa, and the person will end up with a service gap because we didn’t communicate well together.* (Excerpt 6_Actor 5) *Of course, since we’re the only association*, ***if we want to be sustainable, we need to be a little more involved in the contacts (research project coordinator) has with the people in charge of the various programs*** [. . .]. ***But I think we need to be more recognized as an association***, *with precisely these people in charge* [. . .]. (Extract 7_Actor 6)
	Continuity	*There are several elements that need to be worked on, and for that, in my opinion, higher-level coordination is required.* ***For the time being, it really rests on the shoulders of the research team, but ultimately we need to ensure this continuity, and then it needs to be supported by a PDGA, it needs to be supported by a much higher hierarchical level to ensure a good continuity, cohesion and complementarity of service offerings.*** (Extract 8_Actor 3)
Shared definition of diabetes	Outside the box	* Of course, there are many stages, because we have 17 environments with variable geometry, and we don’t go in the same direction as the doctors, so when we want to go down a trajectory, then a way of doing things, well there’s this complexity, it may makes sense for us who are in the public sector, so we manage as we think, but when we want to go down in an environment where there is still private management*, ***it has to make sense for the doctor***. *You know, it’s all well for me to say, “We’ve had the CISSS (region name) ratify such a trajectory for a diabetic patient,” but if he’s* ***stubborn and says, “My pre-diabetics go through the nurse first,” well, that’s difficult.*** *So, it really takes change management, environment by environment, to be able to do it.* (Extract 9_Actor 11) [. . .] *precisely in the transition, when we wanted to work together, but our first project wasn’t working*, ***I appreciated the listening because we were always looking at the nursing angle, and then the nursing angle wasn’t working***, *we were in a cul-de-sac, I felt stuck in a cul-de-sac with the nursing angle, because my nurses could disappear at any time, so having the research team listen to me, having* ***the research team open up to me***, *saying “* ***can we work on something else or work on something differently****? ” For me, that was a great piece of listening between us, which was favorable to the project*. (Extract 10_Actor 5)
	Need for social and community support	*You know, in (city name), we’re not very, very well served in terms of physical activities, I’m trying to find examples, but we don’t have a municipal swimming pool, we lack a lot of things in (city name) to do sports, for seniors, but there are day centers, community centers*, ***do they know all that, day centers, community centers to meet people, to socialize****?* ***A diabetic isn’t just someone with a sweet tooth***, ***he’s someone who needs to talk to others***, ***who needs to socialize*** [. . .], *the psychological aspect is important, so socialization, physical activity, etc.* (Extract 11_Actor 7)
	Broader, more efficient definition	*In fact, it’s that I don’t feel rooted in a common need, I feel part of a research project that identifies relevant things, but I don’t feel mobilized toward a common problem. Then* ***the common problem is perhaps simply the quality of services for people living with diabetes***, *so I say to myself “OK, maybe this is a special moment for me.”* (Extract 12_Actor 13) *So I think that for doctors, it certainly helps them a lot, and* ***it also helps them to better manage patients***, *so that the* ***results are ultimately better than if there was no collaboration***. [. . .] *I* ***believe 110% that if patients are followed up by a nurse every 3 or 6 months, the results are really better***. *You know, we saw when we came back from the pandemic, there were appalling glycemic hemoglobin, here in the morning, I’ve just met a patient for the first time who hasn’t had any follow-up for a year and a half, but I can’t wait to see his hemoglobin, you know, I think it’s going to be disastrous, if he hasn’t taken a blood glucose test*, versus *if he’d had follow-up with a nurse since 2018, taken care of, regular it’s certain that we’d have had better results.* (Extract 13_Actor 8)
	Application to other chronic diseases	[. . .] ***We’re getting out of the box here***, *but it’s to show the extent to which something that’s designed to map out the diabetes trajectory has led us, as a facility, to think about putting together a new offering to meet a need that we’ve come to identify in the course of these discussions, which stem from this project, and which* ***opens up a whole host of other things***. *Because it’s* ***not just diabetes, it’s chronic disease, and what’s good for diabetes is good for chronic disease*** [. . .]. (Extract 14_Actor 4)

The principal researcher’s diary entries highlighted the evolution of the actors’ definition of diabetes throughout the implementation process of the INMED-COMMUNITY pathway, moving from a disease-centered approach to one focused on the needs of the person living with diabetes. The PAR approach led each actor to “step outside the known box,” as one primary care manager put it during the interviews (Table 5, extracts 9 and 10). As one professional stated, the person living with diabetes “needs social and community support” that goes beyond the vision of the disease and care offered by the healthcare system (Table 5, extract 11). A patient partner participating in the INMED-COMMUNITY pathway expressed this and emphasized that collaborations with the community sector would help patient get better health education and information about how to care about their different needs, such as how to manage cooking and alimentation (Table 3, extract 14). Most participants mentioned that development of the INMED-COMMUNITY pathway had the potential to offer a “broader and more efficient” range of services for people living with diabetes, as stated by one professional (Table 5, extract 13). Finally, one of the managers stated that by mobilizing around diabetes, there was potential to adapt the approach to the monitoring of “other chronic diseases” (Table 5, extract 14).

### Alignment of actors, resources, and network support

Notes from meetings have shown that creation of new tools has enabled commitment to mobilization in the INMED-COMMUNITY pathway. These notes also show that each action further developed establishment of working “committees” (Table 6, extract 2),^21^ which were useful in resolving controversies. In fact, there were several mentions of mobilization to “look for new resources,” “validate project orientations,” and develop new tools, such as the “referral form” for diabetics to the community or the “resource directory” (Table 6, extracts 1, 2, 3, and 4). According to a manager and a patient-partner, these actions have enabled a development of a follow-up over time and established “both formal and informal modes of operation” (Table 6, extract 5), partnerships and flexibility in actively responding to controversies (Table 6, extract 6).

**Table 6.** Mobilization.

Theme	Subtopics	Verbatim
Alignment of actors, resources, and network support through the creation of tools	Searching for new resources	*You know, the work (patient-partner) did at the very beginning, with all* ***her research into organizations that could help***, *yes there are things that are relevant in the work she did, and then I think that before the holidays, we all realized that there might be organizations that don’t need to be properly linked to the world of diabetes.* (Extract 1_Actor 6)
	Validation of project orientations	*So, the various meetings are always about getting the various actors involved, then moving the deliverable forward, then always having validations. Often*, ***the big committees are used to validate***, *to say “OK, finally, here’s the process, here’s what we suggest, what do you think?,” and then there’s still room for modification until we say “OK, that’s good enough, let’s put it into action.”* (Extract 2_actor 10)
	Referral form	***For the referral form to XX***, *in fact it was to community organizations, we had a committee, the main committee delegated perhaps half of the people to make it easier* [. . .]. *So, we had a preparatory consultation process, we had a committee that, ultimately, decided in favor of just one form for (name of association), which would then assume the role of a conduit to the other community organizations, so that it doesn’t require everyone to know all the resources, when we see that there’s someone who needs community resources, we refer them* [. . .] *so we’ve clarified that.* ***Now it’s deployed, we have monitoring indicators, and we’re going to track progress with people, with a user guide*** (Extract 3_Actor 10).
	Resources directory	*Yes, I participated in. . . there are various committees that have been set up, more in the community trajectory, so committees work on* ***the resources directory***, ***it’s a tool that was developed, suggested by a patient partner, which brings together resources that would be interesting for diabetic patients according to their needs.*** (Extract 4_Actor 9)
	Informal setting for meetings	*Whatever the case, all these meetings, the way they were conducted, even if they were in Teams, there was a sort of setting that was sufficiently. . . I don’t dare say “loose” there*, ***but that’s a bit it, an informal setting that allowed a lot of exchange***, ***that left room for people to talk***, *to express themselves.* ***There was an interest and curiosity on the*** *part of the team, and all the research people as well, which helped establish that, because we weren’t just saying “well, we’ve got an agenda, then we’ll present something,”* ***we were much more in a spirit of exchange, then collaboration***. (Extract 5_Actor 4)
	Flexibility to respond to controversies	*When we started meeting with people from CLSCs and FMG*, ***I began to see that this was a problem that everyone was aware of***, *and as we went along, participating in these meetings* ***made me start to think differently too***, *I thought, “how can I help them, how can I make my collaboration useful? Even though I was at the meetings, sometimes it’d bothered me at home, and then I’d say to myself, “there must be ways we can work together.”* ***It kind of sparked in me a need to do more*** (Extract 6_Actor 2).

## Discussion

The contribution of our analysis broadens the current understanding of intersectoral collaboration with regards to the complexity of care for people living with diabetes.

The results showed that the actor-network theoretical approach to health intervention in intersectoral collaboration was favorable in supporting a people-centered care for people living with diabetes. In the light of ANT, results showed growth through controversies, contribution of key mediators (project coordination) and shared commitment of human and non-human actors (creation of tools) to engage in an intersectoral collaborative health intervention.

### The importance of profit-sharing through project coordination

The results of our project have shown the importance of project coordination in the process of developing intersectoral collaboration. It played an essential role in mediating power relations and supporting progress in cross-sector action. Coordination facilitated exchanges and transfer of knowledge within the network, while also maintaining an appropriate work pace. Trust was created to commit to a common vision supported by the project team. These results support observations by Borvil et al and Christensen et al recognizing the importance of the role of mediators and their recognition at the level of the socio-technical network.^50,51^

Nonetheless, interactions also generated power dynamics where coordination of actions was important. Certain asymmetrical positions between actors from different sectors generated latent conflicts which impeded progress. Research has identified the risks of focusing on sectoral issues and outcomes that can generate unequal power relationships between actors when seeking cross-sectoral collaboration. For this reason, it is essential that the public policies implemented are not perceived as a burden and that power is shared.^50,52,53^ As this project is in progress, several issues have remained, for which no resolution could be found. Callon^22^ and Latour^25^ state that controversies in the translation process can encourage the enlistment of new participants, stabilize uncertainties, and develop interactions in the network through expression of contradictory arguments and viewpoints. Ultimately, controversies are at the foundation of the network, and our understanding show that their presence enable the network to build, make and unmake itself.^22,25^ Our article thus advances the literature on this subject, going further than Chiari et al^54^ who argued about the importance of integrating actors and resources to advance action. Indeed, our results underline the importance of project coordination as a mediator and facilitator to identify controversies and accompany actors in the progress of action. Considering and identifying controversies was a strength of the project, which quickly mobilized stakeholders in a promising start to cross-sector collaboration. However, as the project moves forward, it will be essential to assess the perceived effects of these actions and gain a better understanding of the implementation process. A subsequent article will describe transitional results seen in the cross-sector partnership actions as part of the INMED-COMMUNITY pathway.

### Mobilizing stakeholders around a common definition of diabetes

Our results show that the formal and informal meetings held during the various phases of participatory action research have helped mobilization and co-creation of network tools. Stakeholders have been able to learn collaboratively around issues for people living with diabetes. As Hendriks et al,^55^ van Eyk et al,^56^ and Chiari et al^54^ all point out, these actions facilitate the integration of stakeholders in the implementation of cross-sector collaboration. All stakeholders take a stand, share different opinions, improve their knowledge, and reframe the problem in such a way that they have an influence on different sectors. This way of considering diabetes as a multidimensional condition has enabled us to move from a disease-centric to a person-centric approach. Furthermore, this understanding of diabetes addresses the complexity of the network and the integration of common ties without compromising the multiple layers of relationships, practices, and meanings specific to each community or group.^57^ In this way, the aim is not to find a standard definition unequivocally adopted, but to remain in a state of questioning, which will continue through the resolution of controversies.^57^ Networks must enable cross-sector partnerships, which allow stakeholders to define and share roles organically, while furthering different perspectives, essential in the creation of a broad, innovative network.^58^

### PAR as a facilitator in the implementation of an intersectoral collaborative health intervention for the follow-up of people living with chronic diseases

The PAR process used to implement the INMED-COMMUNITY pathway facilitated the application of ANT.^34,38^ The interdependence of the network and actors mobilized facilitated the co-construction of the INMED-COMMUNITY pathway and was of great interest for the application of ANT. In fact, ANT functions as a collaborative lever for flexible partnerships. It enables the operationalization of an innovative project by adapting to the context and adopting a holistic approach in response to a complex health situation. PAR combines research and action to produce knowledge that can inform healthcare practices, services, and organizations.^34^ When all team members can act as decision-makers with academics throughout the research process, the likelihood that the results will be relevant and used by these members is increased and therefore beneficial beyond the research objectives.^34,35,59^

Patient partner participation in the PAR process of the INMED-COMMUNITY pathway is also a contributing factor to the success of the intersectoral intervention. The patient partner acted as a bridge between community and health sectors by centering the objectives of the INMED-COMMUNITY pathway on a more “patient-centered approach” and by enabling the project to consider the needs of people living more broadly with diabetes. Hence, the patient partner position in the PAR process of the INMED-COMMUNITY pathway is not limited to its contribution as a patient but involve a form of citizenship where their whole experience as a person living with diabetes is mobilized to make organizational changes on how different sectors communicate and work together.

### Limits

This research has its limitations. First, the fact that the interview guide did not specifically ask actors about controversial issues limited the production of this data in interviews. However, the principal researcher’s field notes and diary highlighted the importance of controversies in the process of intersectoral collaboration. Second, only semi-structured interviews were coded, restricting the rigor of the research; however, these results were triangulated with data from the meetings and the principal researcher’s logbook (XX).^47,49^ Third, the representation of participants from the community sector was limited to 3 stakeholders. This may have skewed the emphasis toward the perspective of the health sector in this study’s results. Finally, this study was carried out in an urban town in Quebec and may therefore not be transposable to other contexts in Quebec, Canada or internationally, thus limiting the scope of the research. Nevertheless, the complexity of the research justified the mobilization of semi-structured interviews, and saturation of the themes was reached during analysis, as no new code emerged.^47^

## Conclusion

This study represents an important contribution to studies of intersectoral collaboration in healthcare, as it broadens current understanding of the complexity of care for people living with diabetes and helps describe the conditions necessary to implement intersectoral collaboration in primary care. The presence of project coordination facilitates involvement in an intersectoral collaborative healthcare intervention. The early recognition of controversies by an accepted and recognized coordinator facilitates negotiation between all stakeholders, enabling commitment to action. The use of these approaches will be considered in the future to facilitate operationalization of intersectoral collaborative health interventions and to measure effects on the health of people living with chronic diseases. Furthermore, future studies of intersectoral collaborative health interventions should examine the effects produced on people’s health data and could explore if the PAR approach and the involvement the community sector can have a significant impact on health results and social determinants of health in people living with chronic illness.

## Appendix 1

### Interview guide for stakeholders

#### Your role

- Tell us about the role you played in developing the community trajectory?
- What is your role in the organization?
- On which committee did you participate?
- What was your role in the committee?
- How did your role differ from that of other participants?
- To what extent do you feel you were involved in all stages of the project carried out by the committee?
- Were you able to use your skills and resources to contribute to the project/activities of the committee?

#### Consideration of points of view

- To what extent do you feel that the points of view of the various partners have been taken into account?
- To what extent do you feel that your point of view has been taken into account?

#### Nature of established collaborations

- In your opinion, were any partnerships established during the project activities?
- In your opinion, what are these partnerships and what do they consist of?
- What new partnerships have you established? What do they consist of?
- What enabled you to establish these partnerships?
- What helped you recognize the contribution that this partner could bring to your business?
- How did you mobilize these partnerships?
- How did you remobilize this partnership outside the project activities?

#### Impact

- What do you see as the impact of these partnerships?
  - For you
  - For other partners
  - For the CISSS Laval organization
- Have you been able to exchange ideas with others to broaden your scope of action?
  - To resolve differences
  - To collaborate differently in the interest of patients
  - To develop new solutions
- Do you intend to maintain these partnerships over the long term? How do you intend to keep them going?
- Do you perceive a change in your role in relation to these partners/partnerships?
- To what extent have you been able to develop your skills or acquire new knowledge through the project? the implementation of new partnerships?
